# Once in a Blue Moon, a Very Rare Coexistence of Glutaric Acidemia Type I and Mucopolysaccharidosis Type IIIB in a Patient

**DOI:** 10.29252/ibj.24.3.201

**Published:** 2019-11-27

**Authors:** Mohammad Reza Alaei, Meghdad Kheirkhahan, Saeed Talebi, Elham Davoudi-Dehaghani, Mohammad Keramatipour

**Affiliations:** 1 Department of Pediatric Endocrinology and Metabolism, Mofid Children's Hospital, Shahid Beheshti University of Medical Sciences, Tehran, Iran;; 2 Department of Medical Genetics, School of Medicine, Tehran University of Medical Sciences, Tehran, Iran;; 3 Department of Medical Genetics and Molecular Biology, Faculty of Medicine, Iran University of Medical Sciences, Tehran, Iran;; 4 Department of Molecular Medicine, Biotechnology Research Center, Pasteur Institute of Iran, Tehran, Iran

**Keywords:** Genes, Iran, Mucopolysaccharidoses

## Abstract

**Background:**

GAI and MPSIIIB are two rare genetic disorders caused by pathogenic variants in two different genes. Here, we report a coexistence of these two different rare disorders in an individual.

**Methods:**

A four-year-old Iranian boy born to first-cousin parents suspected to have MPSIIIB and/or GAI was investigated in this study. Targeted genomic enrichment and NGS were used to examine genes related to MPS and GA. Sanger sequencing was performed to confirm the results.

**Results:**

Two homozygous likely pathogenic variants in *NAGLU* and *GCDH* genes were found and confirmed in the proband.

**Conclusion:**

A combination of specific features of two different diseases in a patient has been reported here. More studies on this case and similar cases can provide more information about the effect of simultaneous pathogenic variants in different genes.

## INTRODUCTION

Glutaric acidemia type I is a rare inherited organic disorder, with a worldwide prevalence of approximately 1 in 100,000 infants. GA1 results from a mitochondrial matrix enzyme deficiency, called glutaryl-CoA dehydrogenase, which is involved in lysine and tryptophan metabolism. Glutaryl-CoA dehydrogenase is encoded by the *GCDH* gene, and its function can be affected by pathogenic variants in this gene. The clinical manifestations of this metabolic disorder can vary considerably even among affected members of a family. Macrocephaly, progressive movement problem, and encephalopathy are some clinical manifestations of GA1^[^^1^^,^^2^^]^.

MPSIIIB or Sanfilippo syndrome type B is another rare metabolic disorder that affects central nervous system. The worldwide prevalence of MPSIIIB is estimated to be between 1/1000000 and 9/1000000^[^^3^^]^. MPSIIIB is caused by the deficiency of the lysosomal enzyme NAGLU required for degradation of heparan sulfate. Recessive pathogenic variants in the *NAGLU* gene are responsible for MPSIIIB. Clinical symptoms of this lysosomal storage disease such as progressive neurodegeneration and mild skeletal changes usually develop between the ages of one and three years^[^^4^^,^^5^^]^. 

Until now, coexistence of two different disorders in the same individual has been reported in different cases, but such phenomenon is hardly observed in case of very rare inherited diseases ^[^^6^^]^. Here, we report a very rare case of coexistence of GA1 and MPSIIIB.

## MATERIALS AND METHODS

**Fig. 1:**
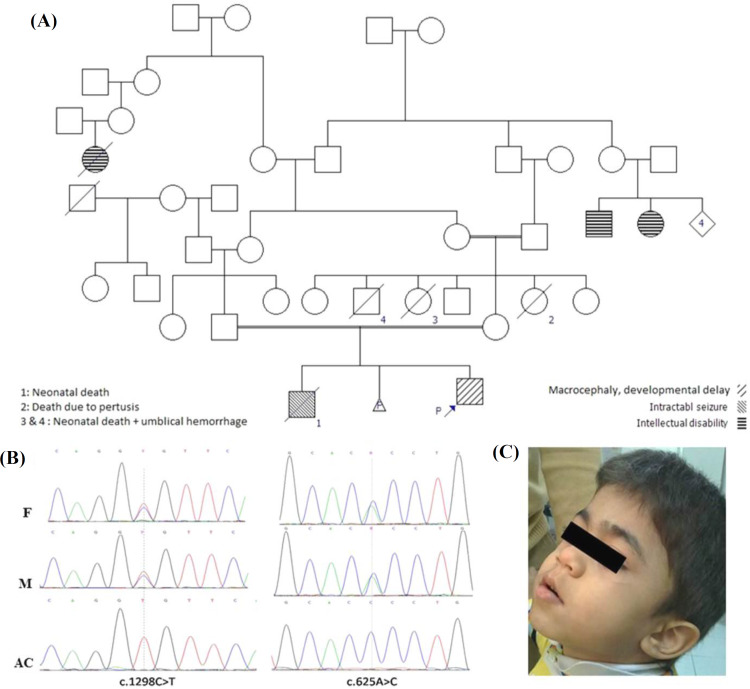
(A) Pedigree of proband's family. Arrow indicates the proband. (B) c.1298C>T and c.625A>C variants in the father (F), mother (M), and the affected child (AC). (C) Facial features of the proband at the age of eight years

A four-year-old Iranian boy born to first-cousin parents was admitted with macrocephaly, developmental delay (head lifting at eight months, sitting with no support at 10 months, crawling at 15 months, and walking at 17 months), speech problem, and the complaints of hyperactivity (Fig. 1). His older brother presented with preterm birth, repeating seizure, and lung problem and had died at neonatal age. Parents of proband completed a questionnaire. 

Bilateral arachnoid cysts in the temporal lobes with extension to Sylvian fissure and increased subarachnoid space over the convexities had been found in this case at five months old. According to the proband’s medical records, the number of his spoken words had decreased after a trauma at 14 months. Concentration problem, falling down when walking, turricephaly, hepatomegaly, wrist weakness, prolonged diarrhea, broad nasal bridge, low-set ears, short palate, macular edema, and short Achilles tendon were other difficulties of this case (Table 1). The medical history and results of biochemical tests (Table 1) provided clues pointing to MPSIIIB and GAI. Genomic DNA was extracted from peripheral blood samples of the proband and his parents using Exgene Blood SV mini kit (GeneAll Biotechnology Co., Ltd., Seoul, Korea) according to the manufacturer's instruction.

**Table 1:** Medical history of the proband

**Medical history items**	**Status**
Prenatal	Progesterone injection due to spotting until 8^th^ week
Birth	NVD, PROM, Turricephaly, BW: 3400g, H: 50 cm, HC: 34.5 cm
Neonatal	Neonatal jaundice
Developmental	GDD (non-progressive Motor delay, Language regression after trauma)
Growth	Height and Weight: NL; Head: macrocephaly; Present (W = 26 kg, H = 128.5 cm)
Familial history	Consanguine parents (first cousin), Boy sibling neonatal death due to intractable seizures
Taking medication	Previous:Carnitin, B2, Resperidon, CoQ10, Zinc, Calcicare, Fluoxetin; Present: Soyfem
Symptoms and signs	Coarse facies, ADHD, Non-progressive GDD, Macrocephaly, Hepatomegaly, Turricephaly
Laboratory findings	MPS (U): {1^st^ :Neg 2^nd^ :Positive}, AA-HPLC-P:{Gln(P): High, ABA: Low}, Metabolic screen (TMS): {Glutarylcarnitine: High},[BS, Na, K, Urea, Cr, NH3, Ca, P, Bilirubin, Total Protein, Albumin, LDH, CPK]: NL, TFT:NL, [SGOT, SGPT, ALP]: NL, AA-TLC:NL, Urine Organic acid GC-MS):NL, CBC:NL; U/A:NL α-N-acetyl-glucosaminidase : Low (<1 nmol MU/mg plasma); Iduronate-2-sulphatase:NL; α-L-Iduronidase: NL; N-Sulphoglucosaminesulphohydrolase:NL; β-Galactosidase:NL
Other findings	MRI (34 m): Bilateral arachnoid cyst in temporal lobe + Periventricular& Subcortical T2 signal; Bone age:NL, Brain CT: Arachnoid cyst+mild atrophy, ABR:NL, EEG: Abnormal (Scattered sharp-spike & slow waves), Acoustic reflex test + Tympanometry (36 m): NL; Fundoscopy: Macular edema; Echo: Thickness of MV & AV+ mild MR and Moderate AI

NVD, normal vaginal delivery; PROM, premature rupture of membranes; BW, birth weight; H, height; HC, head circumference; GDD, global developmental delay; NL, normal; W, weight; ADHD, attention-deficit/hyperactivity disorder; TMS, tandem mass spectrometry; ALP, alkaline phosphatase; SGOT, serum glutamic oxaloacetic transaminase; SGPT, serum glutamic pyruvic transaminase; AA TLC, amino acid thin layer chromatography; GC-MS, Gas chromatography–mass spectrometry; CBC, complete blood count; ABR, Auditory brainstem response

Proband's sample was subjected to targeted genomic enrichment with Nimblegen chip in 14 genes (*GCDH*, *ETFA*, *ETFB*, *ETFDH*, *ARSB*,* GALNS*, *GLB1*, *GNS*, *GUSB*, *HGSNAT*, *IDS*, *IDUA*, *SGSH*, and *NAGLU*) and to examine genes related to MPS and GA. Targeted genomic enrichment and NGS were performed by BGI Shenzhen (Beijing Genome Institute, Shenzhen, China). Sanger sequencing was performed to confirm the existence of identified variants in the proband and his parents. Sequences of primers are not shown but are available upon request. Variant pathogenicity prediction was performed using SIFT, Polyphen-2, and CADD^[^^7^^-^^9^^]^.

**Ethical statement**

The above-mentioned sampling protocols were approved by the Research Ethics Committee of Tehran University of Medical Sciences (IR.TUMS.REC. 1395.2701). Parents of proband signed an informed consent form.

## RESULTS

Two variants NM_000263.3: c.625A>C (NP_000254.2: p.Thr209Pro) and NM_000159.3: c.1298C>T (NP_000150.1: p.Ala433Val) were found in the *NAGLU* and *GCDH* genes, respectively by the NGS. Sanger sequencing confirmed the presence of identified variants in the proband and showed that both parents are heterozygous for these changes. 

*In silico* studies showed that converting a threonine into a proline residue with different size, charge, and hydrophobicity-values at position 209 on the surface of the NAGLU protein can result in the loss of hydrogen bonds and/or can disturb the correct folding. These studies also suggested that replacement of an alanine with a valine residue at position 433 on the surface of the GCDH protein can disturb interactions of this protein with other molecules or other parts of the protein because of different properties of these residues. These findings led to the confirmation of the coexistence of GAI and MPSIIIB in the proband.

## DISCUSSION

So far, a number of studies have been performed to investigate phenotypic heterogeneity of various IMDs but reports on the coexistence of two different IMDs are rare. The study of the complexity of signs and symptoms related to two different metabolic diseases can be helpful to get new insight into the pathophysiology of these groups of disorders. Variant analysis in this study led to the identification of p.Thr209Pro and p.Ala433Val in the *NAGLU* and *GCDH* genes, which can describe the observed combination of phenotypes related to MPSIIIB and GA1 diseases in the proband (Table 1).

The results of *in silico* studies, absence of c.625A>C and an extremely low frequency of c.1298C>T in population databases (0.00002 in GnomAD), the GA1- and MPSIIIB-specific phenotypes in the proband, and co-segregation of the identified variants with the GA1 and MPSIIIB in the family, as well as different reports of these variants in other patients can provide evidence of the pathogenicity of these variants^[^^10^^-^^12^^]^. Therefore, according to the ACMG guideline, both p.Thr209Pro in the catalytic domain of the NAGLU protein and p.Ala433Val in the C-terminal domain of the GCDH protein can be classified as likely pathogenic^[^^13^^]^. 

Genetic studies on the MPSIIIB are very limited in Iran. Until now, only five different pathogenic variants have been reported among Iranian population^[^^14^^-^^17^^]^. Identification of seven different pathogenic variants in six unrelated Iranian MPSIIIB patients can show genetic heterogeneity of this disorder in Iran. Up to the present, more studies have been carried out on the GA1 disease than the MPSIIIB in Iran. The variant p.Ala433Val is one of the reported pathogenic variants in Iranian GA1 patients^[^^11^^,^^18^^,^^19^^]^. 

Given that most of the IMDs are inherited in an autosomal recessive pattern, incidence of metabolic disorders in populations with a high frequency of consanguineous marriages is usually higher than that of other populations. Iran is a country in the Middle East with a high rate of consanguinity. The prevalence of different autosomal recessive disorders in this country can be higher than the worldwide prevalence because of high inbreeding value in this population^[^^19^^]^. 

 In this paper, the coexistence of two different rare disorders in an individual is reported. Further studies on genotype-phenotype correlation in these cases can be helpful for understanding the mechanism by which two pathogenic variants in different genes can lead to multiple phenotypes.

## References

[1] Goodman SI, Markey SP, Moe PG, Miles BS, Teng CC (1975). Glutaric aciduria; a “new” disorder of amino acid metabolism. Biochemical medicine.

[2] Lindner M, Kölker S, Schulze A, Christensen E, Greenberg CR, Hoffmann GF (2004). Neonatal screening for glutaryl-CoA dehydrogenase deficiency. Journal of inherited metabolic disease.

[3] Rare diseases are rare, but rare disease patients are numerous.

[4] Meikle PJ, Hopwood JJ, Clague AE, Carey WF (1999). Prevalence of lysosomal storage disorders. JAMA.

[5] Zhao HG, Li HH, Bach G, Schmidtchen A, Neufeld EF (1996). The molecular basis of Sanfilippo syndrome type B. Proceedings of the national academy of sciences of the United States of America.

[6] Abiri M, Talebi S, Uitto J, Youssefian L, Vahidnezhad H, Shirzad T, Salehpour S, Zeinali S (2016). Co-existence of phenylketonuria either with maple syrup urine disease or Sandhoff disease in two patients from Iran: emphasizing the role of consanguinity. Journal of pediatric endocrinology and metabolism.

[7] Kumar P, Henikoff S, Ng PC (2009). Predicting the effects of coding non-synonymous variants on protein function using the SIFT algorithm. Nature protocols.

[8] Adzhubei IA, Schmidt S, Peshkin L, Ramensky VE, Gerasimova A, Bork P, Kondrashov AS, Sunyaev (2010). A method and server for predicting damaging missense mutations. Nature methods.

[9] Kircher M, Witten DM, Jain P, O'Roak BJ, Cooper GM, Shendure J (2014). A general framework for estimating the relative pathogenicity of human genetic variants. Nature genetics.

[10] Ji RR, Hutchinson A, Jain N, Forbes CD (2018). N-acetyl-alpha-D-glucosaminidase deficiency compositions and methods.

[11] Busquets C, Merinero B, Christensen E, Gelpí JL, Campistol J, Pineda M, Fernández-Alvarez E, Prats JM, Sans A, Arteaga R, Martí M, Campos J, Martínez-Pardo M, Martínez-Bermejo A, Ruiz-Falcó ML, Vaquerizo J, Orozco M, Ugarte M, Coll MJ, Ribes A (2000). Glutaryl-CoA dehydrogenase deficiency in Spain: evidence of two groups of patients, genetically, and biochemically distinct. Pediatric research.

[12] Baradaran M, Galehdari H, Aminzadeh M, Azizi Malmiri R, Tangestani R, Karimi Z (2014). Molecular Determination of Glutaric Aciduria Type I in Individuals from Southwest Iran. Archive of Iranian medicine.

[13] Richards S, Aziz N, Bale S, Bick D, Das S, Gastier-Foster J, Grody WW, Hegde M, Lyon E, Spector E, Voelkerding K, Rehm HL (2015). ACMG Laboratory Quality Assurance Committee. Standards and guidelines for the interpretation of sequence variants: a joint consensus recommendation of the American College of Medical Genetics and Genomics and the Association for Molecular Pathology. Genetics in medicine.

[14] Bunge S, Knigge A, Steglich C, Kleijer WJ, van Diggelen OP, Beck M, Gal A (1999). Mucopolysaccharidosis type IIIB (Sanfilippo B): identification of 18 novel α-N-acetylglucosaminidase gene mutations. Journal of medical genetics.

[15] Ghodsinejad Kalahroudi V, Aryani O, Houshmand M (2014). Molecular characterization of MPS IIIA, MPS IIIB in Iranian patients. In 12th Congress of Iranian Genetics Society.

[16] Najmabadi H, Hu H, Garshasbi M, Zemojtel T, Abedini SS, Chen W, Hosseini M, Behjati F, Haas S, Jamali P, Zecha A, Mohseni M, Püttmann L, Vahid LN, Jensen C, Moheb LA, Bienek M, Larti F, Mueller I, Weissmann R, Darvish H, Wrogemann K, Hadavi V, Lipkowitz B, Esmaeeli-Nieh S, Wieczorek D, Kariminejad R, Firouzabadi SG, Cohen M, Fattahi Z, Rost I, Mojahedi F, Hertzberg C, Dehghan A, Rajab A, Banavandi MJ, Hoffer J, Falah M, Musante L, Kalscheuer V, Ullmann R, Kuss AW, Tzschach A, Kahrizi K, Ropers HH (2011). Deep sequencing reveals 50 novel genes for recessive cognitive disorders. Nature.

[17] Yassaee VR, Hashemi-Gorji F, Miryounesi M, Rezayi A, Ravesh Z, Yassaee F, Salehpour S (2017). Clinical, biochemical and molecular features of Iranian families with mucopolysaccharidosis: A case series. Clinica chimica acta.

[18] Houshmand M, Aryani O, Pirzadeh Z, Ghasemi F, Salehpour S, Tehrani F (2012). Molecular investigation of glutaric aciduria type 1 in Iran. Iranian journal of child neurology.

[19] Saadat M, Ansari-Lari M, Farhud DD (2004). Consanguineous marriage in Iran. Annals of human biology.

